# g:Profiler: a web server for functional enrichment analysis and conversions of gene lists (2019 update)

**DOI:** 10.1093/nar/gkz369

**Published:** 2019-05-08

**Authors:** Uku Raudvere, Liis Kolberg, Ivan Kuzmin, Tambet Arak, Priit Adler, Hedi Peterson, Jaak Vilo

**Affiliations:** 1 Institute of Computer Science, University of Tartu, J. Liivi 2, 50409 Tartu, Estonia; 2 Quretec Ltd, Ülikooli 6a, 51003, Tartu, Estonia; 3 Software Technology and Applications Competence Centre, Ülikooli 2, 51003 Tartu, Estonia

## Abstract

Biological data analysis often deals with lists of genes arising from various studies. The g:Profiler toolset is widely used for finding biological categories enriched in gene lists, conversions between gene identifiers and mappings to their orthologs. The mission of g:Profiler is to provide a reliable service based on up-to-date high quality data in a convenient manner across many evidence types, identifier spaces and organisms. g:Profiler relies on Ensembl as a primary data source and follows their quarterly release cycle while updating the other data sources simultaneously. The current update provides a better user experience due to a modern responsive web interface, standardised API and libraries. The results are delivered through an interactive and configurable web design. Results can be downloaded as publication ready visualisations or delimited text files. In the current update we have extended the support to 467 species and strains, including vertebrates, plants, fungi, insects and parasites. By supporting user uploaded custom GMT files, g:Profiler is now capable of analysing data from any organism. All past releases are maintained for reproducibility and transparency. The 2019 update introduces an extensive technical rewrite making the services faster and more flexible. g:Profiler is freely available at https://biit.cs.ut.ee/gprofiler.

## INTRODUCTION

Interpretation of gene lists from high-throughput studies needs capable and convenient tools based on most up-to-date data. There are a several functional enrichment analysis tools such as Enrichr (1), WebGestalt (2), Metascape (3), KOBAS (4) and AgriGO (5) suitable for positioning the novel findings against the body of previous knowledge. The landscape of enrichment analysis tools is diverse covering different data sources, species, identifier types and methods. While the majority of services provide mappings to the most widely used knowledge resource Gene Ontology (GO) (6), the selection of other data sources varies between the tools. For example, Human Phenotype Ontology (7) is available in Enrichr, WebGestalt, Metascape and g:Profiler (1–3,8), while mirTarBase miRNA target information is included only in a few tools, e.g. Enrichr and g:Profiler (1,8). There are also services that focus on specific species, e.g. AgriGO provides data mostly about plants (5).

These tools have been implemented in a variety of technical platforms. For example, WebGestalt has a well known web server (2), GSEA is known for its stand-alone application (9), Enrichr, in addition to web service, also has an R package (1). Other tools serve its users across a variety of technical platforms. For example, g:Profiler serves its users via a web client and API, Python and R packages, and is available as a tool for Galaxy platform (10).

The input gene lists of functional enrichment tools originate from a broad range of experimental platforms, each having unique identifier types supported by default. Most of the tools accept only a limited subset of possible identifiers, thus creating an obstacle that the users need to overcome via external tools. This common hurdle is avoided in g:Profiler by automatically detecting and accepting close to a hundred different identifier types, possibly mixed together in the same query. This name mapping functionality is provided also as an independent g:Convert service that has already been incorporated as an interoperability function to several tools (11–13).

The methods used for enrichment analysis vary across different tools. g:Profiler, likewise to Enrichr and WebGestalt (1,2), provides the most widely used over-representation analysis approach that uses the hypergeometric test to measure the significance of functional term in the input gene list. There exist tools that provide other methods that also take into account additional ranking information of gene lists (WebGestalt, GSEA (2,9)) or use prior knowledge from gene regulation networks (WebGestalt (2)). All of these methods have their own limitations and there are no good benchmark data to assess and compare different methods (14). In order to serve the users an easy-to-use and fast enrichment tool, g:Profiler has been focusing on one approach only.

**Figure 1.**
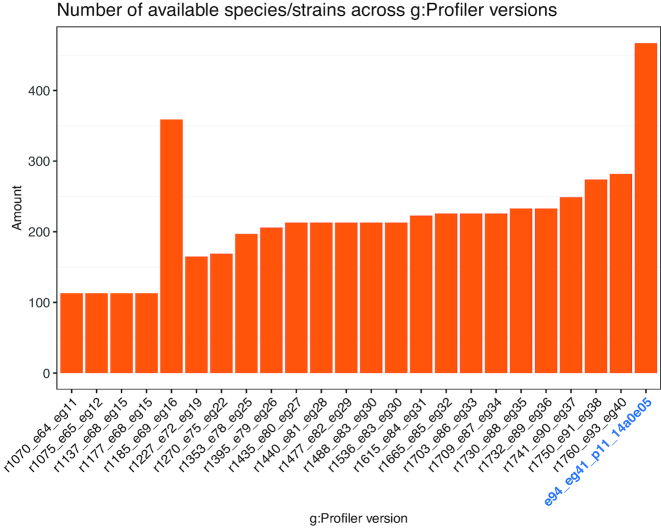
The growth of available species/strains in g:Profiler since 2012. Each column represents an archive version, the current version is highlighted in blue. The isolated peak in 1185_e69_eg16 refers to a version where we incorporated bacterial species to g:Profiler but due to differences in the underlying database structure we dropped them again from further releases.

Only very few of the tools dedicated to enrichment analysis have offered continuous and up-to-date service after their initial release. g:Profiler has remained vital since its first publication in the 2007 NAR web server issue, and has published update articles in 2011 and 2016 (8,15,16). In order to continuously support researchers from a variety of different scientific domains, we have increased the number of supported species and gene identifier types, while keeping the data update frequency, programmable access and core high quality data sources in steady manner throughout the years (Figure 1). As the complexity and size of the underlying data has been growing we have now introduced a complete technical rewrite of the g:Profiler. This allows us to serve our users faster and more conveniently through modern user interface and programming interfaces as well as opens up new avenues for adding features and maintaining the stable service.

## G:PROFILER WEB SERVER

g:Profiler (https://biit.cs.ut.ee/gprofiler) is a collection of tools that are commonly used in standard pipelines of biological entity (gene/protein) centered computational analysis. g:GOSt performs the functional enrichment analysis of individual or multiple gene lists, g:Convert maps gene/protein identifiers between various namespaces, g:Orth allows to map orthologous genes across species and g:SNPense maps human SNP identifiers to genes.

### g:GOSt – functional enrichment analysis

g:GOSt is the core tool for performing functional enrichment analysis on input gene list. It maps a user provided list of genes to known functional information sources and detects statistically significantly enriched biological processes, pathways, regulatory motifs and protein complexes. Main source of information about genes, identifier types and GO terms and associations is the Ensembl database (17). The Gene Ontology is the most comprehensive resource in g:GOSt covering 467 species and strains represented in Ensembl Genomes and fungi, plants or metazoa specific versions of Ensembl (17), and parasite specific data from WormBase (18).

We include most common data sources that are also regularly updated. This covers currently KEGG (19), Reactome (20) and WikiPathways (21); miRNA targets from miRTarBase (22) and regulatory motif matches from TRANSFAC (23); tissue specificity based on expression data from Human Protein Atlas (24); protein complexes data from CORUM (25) and human disease phenotype associations from Human Phenotype Ontology (7). Users are free to choose any combination of the provided data sources. This allows users for example to focus either on cellular components from the Gene Ontology or disease information from the Human Phenotype Ontology. For all data sources, except for two (KEGG and Transfac due to the licensing reasons), users can also download all the underlying terms and associations.

The functional enrichment of the input gene list is evaluated using the well-proven cumulative hypergeometric test. For a single gene list, we are testing numerous functional terms at a time. For example, already >16 000 GO biological process terms are considered for a human gene list. To reduce the amount of false positive findings, g:GOSt performs multiple testing correction. By default we use the original g:SCS (Set Counts and Sizes) correction method introduced back in 2007 (15). Unlike the standard methods, g:SCS considers the dependency of multiple tests by taking into account the overlap of functional terms. We repeated the simulations for verifying the g:SCS method and confirmed that the method still holds the same properties. Namely, g:SCS is more conservative than Benjamini-Hochberg False Discovery Rate but not as strict as Bonferroni correction. Instead of the default g:SCS method user can also choose to apply the Bonferroni correction or the Benjamini–Hochberg False Discovery Rate. It is noteworthy, that in g:GOSt we report only the adjusted enrichment *P*-values.

The input gene list can be either unordered or ordered. By default, the list is considered unordered and complete. If there exists some natural ranking of the genes, the ordered gene list option can be used. For example, the fold change in between different biological conditions, the number of neighboring nodes in a protein-protein interaction network, the absolute expression values or any other information relevant for the experimental setup could be a good basis for such ranking. In such cases the hypergeometric testing is performed for each possible prefix of the list starting from the first gene and adding next genes one by one. The smallest enrichment *P*-value is reported for each of the terms along with corresponding gene list length. For different terms this length can vary, especially as the broader terms can be enriched for larger lists only.

The input can also consist of multiple lists simultaneously using a FASTA format like symbol in front of every list. This multi-list analysis feature was previously implemented in the g:Cocoa tool.

By default, g:GOSt uses the set of all annotated protein-coding genes as a background. In some experiments however, only a subset of genes or proteins is measured. For example, the targeted sequencing of only disease specific genes would imply enrichment of that disease association. Statistically, for these cases it is recommended and sometimes necessary to use custom background information when calculating the statistical enrichment significance. The custom background should include a list of genes that were actually measured during the biological experiment, such as all genes in the sequencing panel. This option allows to calculate a more precise evaluation of functional enrichment.

The enrichment results in g:GOSt are first highlighted in a novel Manhattan plot. It is accompanied by a more extensive and interactive results table giving detailed information about every gene and term. Both of these outputs are customizable and downloadable in a publication ready format.

### g:Convert – automatic conversion of gene identifiers

g:Convert is able to convert between various gene, protein, microarray probe and numerous other types of namespaces. We provide at least 40 types of IDs for more than 60 species that we obtain from Ensembl Biomart (26). The most complex are human data where 98 different identifier types are included currently. All identifiers are obtained through matching them via Ensembl gene identifiers (ENSG) as a reference. g:Convert also accepts mixed identifier types as input.

The output of g:Convert is a table with converted IDs, gene names and corresponding gene descriptions. Users can also retrieve the list of genes associated to any terms used in g:GOSt (such as Reactome pathways, HPA or GO biological process category). Other services, such as the Ensembl Biomart (26), or Uniprot mapping service (27) include similar range of identifiers. However, g:Convert accepts a mixture of identifiers and does not demand specifying original identifier type first by the user. This improves the interoperability and helps to connect external services beyond the g:Profiler toolset.

### g:Orth – mapping orthologous genes across species

g:Orth makes use of orthologous gene mapping information from the Ensembl database. It enables the user to retrieve orthologous genes corresponding to their input gene list automatically. The mapping is executed in two-step manner by first converting the input gene IDs provided by the user to Ensembl ENSG identifiers, and then retrieving the corresponding orthologous gene information for target species. g:Orth can be used to transfer the knowledge collected for well studied model organisms to less studied species. Conducting enrichment analysis after orthologous mapping might lead to more comprehensible results than it would be possible when using only the original species.

### g:SNPense – SNP identifier mapping

g:SNPense allows the user to easily map a list of human SNP rs-codes (e.g. rs7961894) to gene names, receive chromosomal coordinates and predicted variant effects. Mapping is enabled for such variants that overlap with at least one protein coding Ensembl gene. All underlying data are retrieved from the Ensembl Variation Data (28). The variant effects are described with color-coded set of variant consequences terms, defined by the Sequence Ontology (29). These terms convey the information about the effects that each allele of the variant may have on each gene.

### Programmatic access

All the tools in g:Profiler web server are accessible in GNU R and Python via dedicated software packages gprofiler2 and gprofiler-official, respectively. These packages enable the community to integrate g:Profiler tools to different automated pipelines or to easily access the results for other custom visualizations. Using these packages one can also improve the reproducibility of the analyses as the parameters will be self-documented in the code. The updated R and Python packages utilize the new JSON APIs that now offer a more standardized way to access g:Profiler programmatically. Moreover, the new R package gprofiler2 provides the same interactive visualizations as the ones available in the web tool.

### Data and software archives

g:Profiler follows a quarterly data release cycle of Ensembl with up to a couple of months lag to update and verify all our underlying datasets. We support data sources that are well established and continuously updated. For the last 8 years we have kept the complete archive of previous data releases and respective software developed up until that moment. This allows users to repeat and validate the analyses without worrying about the changes in data or software that have taken place over the time. During these 8 years we have published 27 database versions and moved from supporting Ensembl version 62 to Ensembl version 94 (see Figure 1).

## NEW DEVELOPMENTS IN G:PROFILER IN 2019

With this update article we have introduced a complete rewrite of the established service. This brings faster and more customizable back-end and modern user interface (Supplementary Figure S1).

### Publication ready results

**Figure 2.**
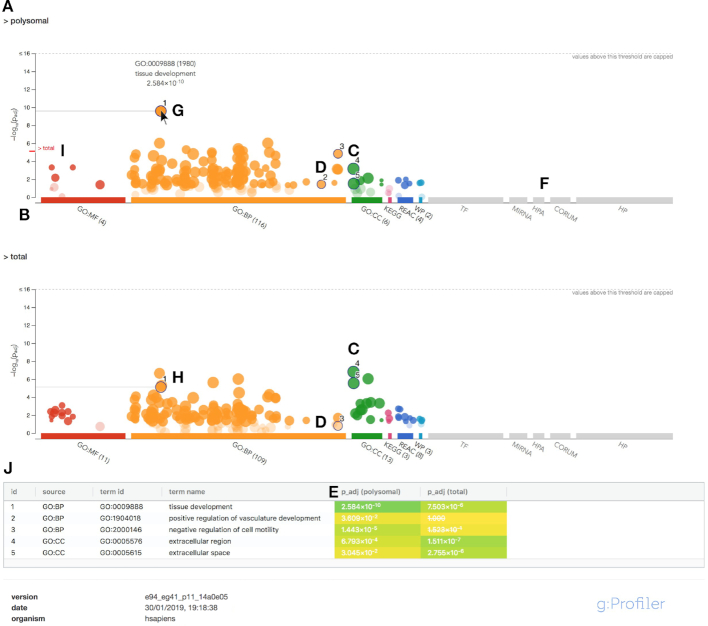
Example of g:GOSt multiquery Manhattan plot. (**A**) Name of the query. (**B**) X-axis shows the functional terms grouped and colour-coded by data source. (**C**, **D**) The position of terms in the plots is fixed and terms from the same (GO) branch are close to each other. (**E**) *P*-values in the table outputs are color-coded from yellow (insignificant) to blue (highly significant). (**F**) Data sources that were not included in the analysis are shown in grey. (**G**) Hovering over the term circle shows a tooltip with the most relevant information. (**H**) In case of a multiquery, the same term is also highlighted on other plots. (**I**) The *P*-values from other queries are indicated in red next to the y-axis for easier comparison. (**J**) A click allows to pin the circles to the plot together with a numeric ID and creates a more detailed result table below the image.

The highlight of the new release is an interactive Manhattan plot, well known from GWAS studies, and used for the first time for enrichment analysis according to our knowledge. g:GOSt returns this plot illustrating the enriched terms across all the analysed term categories (Figure 2). The x-axis shows the functional terms and the corresponding enrichment *P*-values in negative log_10_ scale are illustrated on the y-axis. Each circle on the plot represents a single functional term. The circles are color-coded by data source and size-scaled according to the number of annotated genes in that term. The lighter circles represent insignificant terms and the data sources that were not analysed (omitted by user) are in grey (Figure 2F).

The term location on the x-axis is fixed, data sources are grouped together and colour-coded for faster and more intuitive interpretation of the results (Figure 2B). Terms from the same GO subtree are located close to each other. The fixed location highlights the term groups from a particular GO subcategory as these terms tend to be enriched together forming a peak in the Manhattan plot (Figure 2C). In addition, it enables to easily spot the differences in the enriched terms across several queries (Figure 2D).

Similarly to the previous versions, g:GOSt provides a result table containing information about the enriched terms, overlap sizes and corresponding *P*-values. With this update the results table is made interactive. All the data can be filtered to highlight or limit the results according to a specific keyword or term size. Also, columns in the results table are sortable and their order can be changed by dragging the column headers. In addition, columns of limited interest can be dropped from the view by dragging them away from the table. This allows the user to pick and choose the columns carrying the most relevant information before extracting the data in publication ready image as PNG and as CSV or GEM files suitable for further analysis.

In all the results tables we describe the adjusted *P*-values by an intuitive color-blind friendly viridis colour scheme which runs from yellow representing insignificant findings to dark blue with the smallest possible *P*-values (Figure 2E).

### Flexible input

The input gene list can be composed of any common gene/protein identifiers used by the life science community in a mixed manner. Thanks to g:Convert running in the background the users can provide gene names mixed with protein accession numbers, probeset IDs, chromosomal locations and SNP IDs. This provides the users the freedom to analyse their results straight-away without additional manual steps of transforming their platform specific identifiers to a particular identifier type to get their first enrichment results. Since this 2019 version g:GOSt also accepts as an input the chromosomal intervals in browser extensible data (BED) file format. This makes it more straightforward to integrate g:GOSt with the annotation based browsers like UCSC Genome Browser and many DNA modification analysis tools that output BED files. All the chromosomal locations are mapped to the latest Ensembl Genome version that g:Profiler toolset is built upon. Currently we support the human genome version GRCh38.p12.

### Multiple queries

g:GOSt input can consist of either a single gene list or a set of gene lists. Starting from g:Profiler 2011 version this was possible through a separate g:Cocoa tool. For simplicity and better user convenience, we merged this functionality into g:GOSt. We automatically evaluate the input type and launch the enrichment analysis for all the provided gene lists.

The level of details in the output depends on the number of query lists. In case of a single list, the results table includes evidence codes for each term-gene pair. As the multi query option is useful for comparing multiple enrichment results, the table focuses on comparing *P*-values across those input lists. We highlight the terms that are significant for at least one of the queries and allow an easy color-coded comparison to pick up the most significant terms.

In the multiple list query view, we produce a separate Manhattan plot for each of the input lists. Despite producing separate plots, the resulting images are interlinked. This allows to highlight the same term across multiple queries by hovering over terms in any of the individual plots.

### Data sources

We have changed the underlying data source for the miRNA targets. Previously we used Microcosm Targets prediction service but it is not supported anymore. This motivated us to move to experimentally validated information about microRNA targets and now g:GOSt includes dedicated interaction database miRTarBase as a data source (22).

We have introduced with this update WikiPathways (21) as a complementary pathway data source. WikiPathways is a community driven initiative that shows consistent and steady increase in annotated pathways reaching close to 3000 pathways for approximately 30 organisms.

Since this 2019 version of g:GOSt we have enabled users to use any other relevant data sources by accepting custom Gene Matrix Transposed file format (GMT files). Custom GMT file support enables users to find enriched terms that might be specific for their scientific topic or not regularly updated and therefore not selected as default data source for g:Profiler. This allows us to focus our resources on providing limited but high quality and up-to-date data while providing the flexibility of analysing other species or data to the users.

Users can either compose GMT files themselves or use pre-compiled gene sets available for example at Molecular Signatures Database (MSigDB) (30) or at http://baderlab.org/GeneSets. We also include example GMT files that might be useful for our users, e.g. drug–gene relationships from PharmGKB (31), on a dedicated section of the Documentation in g:Profiler.

### Technical implementation

This release of g:Profiler embodies an extensive update on both the back and front end. The core parts of the toolset have been revised and rewritten in order to keep up with the modern development trends and to speed up calculations. The back end is now reimplemented in Python 3.6 employing the widely used packages such as numpy, pandas, scipy and statsmodels. The biggest change in the back end lies in the data structure for keeping and accessing the annotations. We replaced the previous BerkeleyDB engine with SQLite database and utilised the Roaring bitmaps (32) data structure resulting in faster set operations.

The REST API is developed in Python 3.6 using the Flask framework. The user interface is mainly based on HTML5 and JavaScript and built with the open-source Vue.js framework (https://vuejs.org/). The responsive result tables are created using ag-Grid.js (https://www.ag-grid.com/) library and D3.js (http://d3js.org) library is used for visualization components.

The new implementation is also better modularized making it easier to maintain and introduce new developments in the future.

### Omitted resources

We have removed the g:Sorter functionality that allowed finding similarly expressed genes in transcriptomics data as this functionality is better supported by the Multi Experiment Matrix tool dedicated for gene expression similarity analysis (12).

Due to the licencing changes we stopped providing the Online Mendelian Inheritance in Man (OMIM) data source. We have also decided to remove the Biogrid network visualisation feature as there are better dedicated tools for analysing and visualising such data, e.g. Cytoscape (33).

## USE CASE

To illustrate the several new features in g:Profiler we have used data published by Robert *et al.* (34). They analysed the total mRNA and the mRNA fraction associated with polysomes on human adipose tissue-derived stem cells (hASCs) at 24 h of osteogenesis induction. Differentially expressed genes (DEGs) were identified by paired comparisons between non-induced cells and induced cell conditions, and for polysomal and total RNA fractions. GO enrichment analysis of DEGs from both total and polysomal RNA fractions using previous g:Profiler version was part of their analysis pipeline. The enrichment results were then visualized using REVIGO (35). Most of the biological processes were enriched in both of the groups and related to response to external stimuli, cell communication and development.

We repeated the enrichment analysis with the same lists of DEGs in the updated version of g:Profiler. This use case is a typical example for g:GOSt ordered multiquery analysis that allows to compare the two sets of DEGs ordered by the FDR value. As a result we first see a Manhattan plot for both of the two queries indicated by the query name (Figure 2A).

The details of a term are shown in the tooltip that appears on hover (Figure 2G). The two plots are interlinked so that the corresponding term is also highlighted in the other plot (Figure 2H) and the *P*-value is shown next to the y-axis for better comparison (Figure 2I).

User can select circles to pin by clicking on them. This attributes the circle with an identifier and results in a new row to the descriptive table below the image showing the IDs, names and *P*-values for the selected terms (Figure 2J). Similar but more detailed table appears in the Detailed results tab.

With this use case we want to highlight that users can now obtain publication ready images that illustrate the significant terms together with their *P*-values. Our Manhattan plot visualization can serve as an alternative and more intuitive option for widely used REVIGO visualizations that our users often have used to illustrate the findings provided by g:Profiler.

## DISCUSSION

In this 2019 update of g:Profiler we have introduced a complete technical rewrite of our well established service. The new version of g:Profiler toolset provides faster and more intuitive service while keeping the technical robustness, data quality and data update frequency at the same high quality level as before. Since the initial publication in 2007 the controlled vocabularies (e.g. GO) and knowledge bases (e.g. KEGG) have grown considerably in size and new data sources continue to emerge (WikiPathways, DisGeNET) (21,36). By including data about more species, the underlying data has grown by an order of magnitude requiring us to move towards better suited data structures. This new architecture also made it possible to enable users’ own GMT files to be used for enrichment analysis, a request that our users have asked already for a while. We have also implemented a new responsive graphical user interface to improve the user experience. The customisability of the results tables and highlighting options of the Manhattan plot allow users to prepare publication and presentation ready images straight from the website.

One of the goals of g:Profiler is to continually help scientists to conduct their research in a reproducible and transparent manner. Many web services, including for enrichment analysis, fall into oblivion after the initial release. Therefore the users need to have a critical mind to recognize services that ignore data updates and might miss relevant new findings (37). We prevent this by paying a close attention to data quality and short- and long-term availability.

g:Profiler with its fast and versatile components have been recognized by European Life Science Infrastructure ELIXIR as a Recommended Interoperability Resource to be of fundamental importance to the research infrastructure of the life sciences (https://www.elixir-europe.org/platforms/interoperability/rirs). Our services allow to eliminate an annoying and time consuming task majority of life science researchers still face when trying to use web tools that accept only specific platform identifier types. With g:Profiler toolkit we provide the identifier mapping and orthologous gene conversion services for both the end users and other service providers. The variety of technical platforms that we support, the interactive web application and the JSON API, the R and Python packages, and Galaxy tool, should allow anyone to find a suitable way to incorporate the interoperability services into their everyday practice.

g:Profiler has been taught in several ELIXIR member states, as well as in Canada and USA and plays a central role in a recently published pathway enrichment analysis and visualization of omics data protocol (38). g:Profiler is also a major component of an enrichment analysis based automatic clustering tool funcExplorer that benefits on programmatic access from the g:GOSt service (13).

Future developments of g:Profiler will focus on providing biologically more relevant results to the users. On one hand it needs analysis and adaptation of alternative gene set enrichment methods. Since the functional terms are often not independent from each other, we need methods that are capable of taking into account dependencies between the terms and the overall graph structure of the ontologies. Some enrichment tools, such as the topGO package in R, propose methods that take the graph topology of GO into account while testing the enrichment (39). However, this is a remaining challenge that needs further research. On the other hand, the personalized medicine initiatives generate enormous data sets about SNPs and enrichment analysis is moving from gene level to single nucleotide level when studying associations with traits. Therefore, we aim to expand our g:SNPense tool to make use of the large datasets from GWAS and eQTL analyses and to put their results into context and highlight the statistically significant relationships from there.

## Supplementary Material

gkz369_Supplemental_File
