# Supplementary material for: g:Profiler: a web server for functional enrichment analysis and conversions of gene lists (2019 update)
Source: Nucleic Acids Res. 2019 May 8;47(W1):W191–8. doi: 10.1093/nar/gkz369 (PMC6602461; doi:10.1093/nar/gkz369)
Supplement: gkz369_Supplemental_File [file gkz369_supplemental_file.pdf]

g:Profiler – a web server for functional enrichment analysis and conversions of gene lists (2019 update)

Uku Raudvere, Liis Kolberg, Ivan Kuzmin, Tambet Arak, Priit Adler, Hedi Peterson, Jaak Vilo

Supplementary material

	Feature	g:Profiler 2016	g:Profiler 2019
Data	Number of species/strains	213	467
	Number of ID types for human	116	98
	Total number of ID types	369	429
	Underlying databases	Ensembl and Ensembl Genomes Fungi, Metazoa, Plants	Ensembl and Ensembl Genomes Fungi, Metazoa, Plants, **WormBase ParaSite**
	Data sources	GO, KEGG, Reactome, TRANSFAC, **miRBase**, HPA, CORUM, HP, **OMIM**, **BioGRID**	GO, KEGG, Reactome, **WikiPathways**, TRANSFAC, **miRTarBase**, HPA, CORUM, HP
Techical	Database	Berkeley DB	SQLite
	Main codebase	Perl	Python 3
	Median response time in seconds	13.0215	1.508
	Front-end properties	mostly static	interactive
	API response	CSV	JSON
Options	Visualisations	-	Manhattan plot
	Multiple query lists	g:Cocoa	separate option in g:GOSt
	g:Sorter search tool for gene expression profiles	+	-
	Query input type	list	list, **BED file**
	Custom annotations	-	GMT file
	Export file formats	PNG, CSV, PDF, XLSX, GEM	PNG, CSV, GEM
Packages	R package	gProfileR	gprofiler2
	Python package	gprofiler-official **0.3.5**	gprofiler-official **1.0**

Figure S1: Overview of feature updates and changes in g:Profiler 2019 in comparison to previous update article in 2016.
